# Effectiveness of psychotherapeutic interventions on psychological distress in women who have experienced perinatal loss: a systematic review protocol

**DOI:** 10.1186/s13643-020-01387-6

**Published:** 2020-06-02

**Authors:** Elyse M. Charrois, Katherine S. Bright, Abdul Wajid, Muhammad Kashif Mughal, K. Alix Hayden, Dawn Kingston

**Affiliations:** 1 Faculty of Nursing, University of Calgary, 2500 University Drive, N.W., Calgary, Alberta T2N 1 N4 Canada; 2 Alberta Children’s Hospital Research Institute (ACHRI), Calgary, Alberta Canada; 3 Alberta Health Services, Calgary, Alberta T2S 3C3 Canada; 4 Libraries and Cultural Resources, University of Calgary, T2N 1 N4, Calgary, Alberta Canada

**Keywords:** Systematic review, Protocol, Perinatal loss, Pregnancy loss, Psychotherapy, Psychological interventions, Therapy, Psychological distress, Adjustment, Coping

## Abstract

**Background:**

Perinatal loss is a traumatic and complex experience that contributes to negative maternal psychological states and adverse outcomes impacting fetal development, maternal-fetal/infant bonding, marital/partner relationships, and child cognitive, emotional, and behavioral development. These outcomes present preventable disease burden and financial liability to individuals, families, and the healthcare system. Psychological interventions have the potential to improve outcomes for women and their families after perinatal loss. A few studies have explored the effectiveness of individual psychotherapeutic interventions in reducing maternal psychological distress after perinatal loss; however, a systematic review to compare these interventions has not been conducted. The primary objective of this systematic review is to determine the effectiveness of psychotherapeutic intervention on psychological distress and perception, coping, and adjustment in women who have experienced perinatal loss. The secondary objective of this review is to examine the content and delivery methods of effective psychotherapeutic interventions.

**Methods:**

We endeavor to search electronic databases (PsycINFO, MEDLINE, Embase, Cochrane Central Register of Controlled Trials (CENTRAL), Scopus, CINAHL, Social Work Abstracts, Family and Society Studies Worldwide, Family Studies Abstracts, Academic Search Premier), gray literature databases (Proquest Dissertation and Theses Global, Web of Science Conference Proceedings Citation Index, OAIster, Open-Grey, Canadian Electronic Library, Canadian Research Index), and relevant organizational websites and conduct forward and backward citation searches of included studies. Inclusion criteria will consider studies that (1) are randomized controlled trials (RCTs), quasi-experimental (e.g., before-after design), and observational (prospective cohort); (2) include women affected by perinatal loss accessing psychotherapeutic intervention or support; and (3) evaluate a mental health or related outcome. Two authors will independently screen all citations, full-text articles, and abstract data. The study methodological quality (or bias) will be appraised using an appropriate tool. The primary outcome(s) will be measurements on the severity of depressive, anxiety, grief, and post-traumatic stress symptoms. Secondary outcomes will include measurements on difficulties in perception, coping, social, or dyadic adjustment. Conducting a narrative synthesis will identify relationships within study findings, and if appropriate, a random effects meta-analysis will be performed.

**Discussion:**

This systematic review will summarize the effectiveness of psychological interventions, including their content and delivery method, in reducing psychological distress and improving outcomes for women affected by perinatal loss. The evidence generated from this review can inform researchers and policymakers in expanding on related research and developing customized interventions or programs.

**Systematic review registration:**

PROSPERO CRD42019126456.

## Background

Perinatal loss (PL) can be experienced as a devastating and psychologically distressing occurrence which studies have shown negatively impacts mental, emotional, and physical health across the lifespan [1–5]. Perinatal loss (PL), which includes prenatal loss (miscarriage or stillbirth) or neonatal death, may occur at any time between the point of conception to 28 days after the date of delivery [6–9]. Canadian guidelines define miscarriage as the loss of a pregnancy before 20 weeks’ gestation, stillbirth as death after 20 weeks’ gestation with the fetus weighing over 500 g, and neonatal death as loss of an infant within 28 days after birth [6–9]. Definitions of miscarriage, stillbirth, and neonatal death may vary worldwide because of the lack of standardization.

Across Canada, the USA, and the UK, it is estimated that between 15 and 20% of clinically identified pregnancies result in miscarriage [2, 4, 10–24]. This estimate is higher for those who have previously lost a pregnancy [25], increasing to 75% for women 45 years of age and over [21]. One study suggested that the actual prevalence of miscarriage, including missed or undocumented miscarriages, represents 30 to 40% of all pregnancies each year [26]. These prevalence rates suggest there are many women who have experienced a unique type of loss that is surrounded by various forms of ambiguity [27–29], rendering it particularly traumatizing and difficult to process [28]. Further, perinatal bereavement is considered a complex, emotional and distressed response that has shown to last an indeterminate length of time [30]. Studies have found that perinatal loss (PL) has substantial association with expressions of psychological distress such as depression, anxiety, post-traumatic stress (PTS), eating disturbance, preoccupation with the lost fetus/infant, and sleeping disorders [6, 31, 32]. Despite this association, there is insufficient evidence in the literature that describes and compares psychotherapeutic interventions effective in reducing psychological distress in women after perinatal loss (PL) [33]. Limitations such as these may reinforce women’s reticence in seeking resources to care for their mental and emotional health [34, 35] and health care professional’s enduring exclusion of mental and emotional health assessment from standard perinatal care [36]. However, there are some individual studies that have found psychotherapeutic interventions helpful with reducing psychological distress symptoms in women after perinatal loss (PL) [37–39]. This is especially true for women who are finding recovery from the PL experience excessively challenging [11, 40–42]. It is possible then that some psychotherapeutic interventions are more effective with improving psychological distress in women after PL, than others.

### Psychotherapeutic interventions

While little is known about interventions that are effective with improving psychological distress in women affected by perinatal loss (PL) [33], when asked, most women indicated that they would prefer to be under the care of a therapist to help them cope [43]. As such, a specialized program or licensed therapist or registered psychologist knowledgeable in promoting mental health after PL would have the expertise to assist women in discussing their loss, help them to understand, and regulate their emotions while offering non-judgmental support and resources [34, 44].

In the literature, there are a few intervention studies that provide data on the effectiveness of cognitive behavioral therapy (CBT) [11, 15–17, 23, 40, 45, 46], interpersonal psychotherapy (IPT) [37, 42, 47], bereavement counseling [38, 48], grief therapy [49], and other psychological and supportive interventions or programs [19, 50, 51]. Despite this, a comprehensive comparison of these intervention studies does not exist. This systematic review will identify the psychotherapeutic intervention(s) with the strongest evidence to suggest superior efficacy in reducing psychological distress in women after PL and examine their content and method of delivery. With the knowledge generated, it is intended that the quality of psychotherapeutic services made available and accessible to women after PL will improve.

## Methods

### Objectives

The primary objective of this systematic review is to determine the effectiveness of psychotherapeutic intervention on psychological distress (depressive, anxiety, grief, posttraumatic stress symptoms) and perception, coping, and adjustment in women who have experienced perinatal loss (PL). The secondary objective of this review is to examine the content (structure, objectives, goals) and delivery methods (in-person, telephone, online, distance) of effective psychotherapeutic interventions.

### Review questions

To address the objectives of this systematic review, the following questions will be answered:

1. What is the effectiveness of psychotherapeutic intervention on psychological distress in women who have experienced PL in comparison with women who do not receive psychotherapeutic intervention?
2. What is the effectiveness of psychotherapeutic intervention on difficulties with perception, coping, and adjustment in women affected by PL in comparison with women who do not receive psychotherapeutic intervention?
3. What is the content and delivery method of the psychotherapeutic intervention that is associated with reducing psychological distress and improving perception, coping, and adjustment in women who have experienced PL?

### Protocol and registration

This protocol is being reported in accordance with the reporting guidance provided in the Preferred Reporting Items for Systematic Review and Meta-Analysis Protocol (PRISMA-P) statement (Additional file 1) [52]. This review has been registered in the International Prospective Register of Systematic Reviews (PROSPERO) with registration number CRD42019126456. The proposed systematic review and meta-analysis will be reported in accordance with the reporting guidance provided in the Preferred Reporting Items for Systematic Reviews and Meta-Analyses (PRISMA) statement [53].

### Eligibility criteria

Criteria identifying studies that are eligible are outlined in PICOSS format (participants, interventions, comparators, outcomes, study designs, and setting) as described below [54].

#### Participants

Studies will be included if their participants are female, over 18 years of age, and have experienced any type of perinatal loss as a single or recurrent event. Perinatal loss (PL), which includes prenatal loss (miscarriage or stillbirth) or neonatal death, may occur at any time between the point of conception to 28 days after the date of delivery. Miscarriage is defined as the loss of a pregnancy before 20 weeks’ gestation, stillbirth as death after 20 weeks’ gestation with the fetus weighing over 500 g, and neonatal death as loss of an infant within 28 days after birth [6–9]. A recurrent perinatal loss will be defined as two or more losses occurring consecutively [55]. Studies with participants who are visiting health centers or specialized programs for their perinatal loss or receiving prenatal care for a pregnancy subsequent to a previous perinatal loss will be included. Studies with participants who have experienced an ectopic pregnancy or termination of pregnancy will be excluded.

#### Measurement

Primary and secondary outcomes may be evaluated using a validated and reliable psychometric measurement tool or a validated questionnaire. However, studies that do not use at least one validated psychometric measurement tool will be excluded.

#### Intervention

The intervention received may be a psychotherapeutic intervention that was facilitated through a specialized program, or by a registered psychologist, licensed therapist, or other trained and licensed professional credentialed to provide specific counseling. The intervention may include psychological counseling, psychotherapy, psychological support, and psychoeducation in sessions structured to specific objectives or goals (content), conducted individually or in groups, and facilitated in person, on the telephone, online, or via distance delivery (method of delivery).

#### Comparators

Studies with any type of comparator group will be included. The comparators may represent the group receiving usual care, standard care, routine care, or intervention, another psychological or other non-specific intervention or a group that has been waitlisted.

#### Outcomes

The primary outcomes of interest include measurements on the severity of depressive, anxiety, grief, and post-traumatic stress symptoms that will have been evaluated using validated psychometric measurement tools according to their own clinical cutoff points. High symptom severity identified on psychometric measurement tools may suggest clinical caseness but does not determine a diagnosis. The secondary outcomes of interest include measurements on difficulties in perception, coping, social, or dyadic adjustment. Difficulties in perception, coping, social, and dyadic adjustment are defined within the parameters of the psychometric measurement tool that is being used to evaluate each dimension.

#### Study design

Based on a preliminary scoping search, experimental and quasi-experimental studies are primarily expected, findings from which will be incorporated to address the review questions. Research focused on providing data related to the primary and secondary outcomes of this systematic review may include randomized controlled trials (including pilot randomized controlled trials), quasi-experimental studies (e.g., non/equivalent control group design, single group, pre-test/post-test, or before-after design) and observational prospective cohort studies.

#### Setting

Eligible literature will not be limited by specific setting or geographical location.

### Search strategy

A search strategy was developed and revised by a university-based health librarian (KAH) and the primary author (EMC). The search was developed in PsycINFO and piloted to ensure that all seed studies were retrieved. The PsycINFO search was then translated for each identified database, with subject headings responsive to the database vocabulary, and keywords constant. The databases that were searched from their inception onwards within disciplinary databases included PsycINFO, MEDLINE, Embase, Cochrane Central Register of Controlled Trials (CENTRAL), CINAHL, Social Work Abstracts, Family and Society Studies Worldwide, and Family Studies Abstracts and within interdisciplinary databases including Scopus and Academic Search Premier (Additional file 2). Database searches will be updated prior to submission of the final publication to ensure all new studies are captured. Gray literature will be searched by the primary author within databases including Proquest Dissertation and Theses Global, Web of Science Conference Proceedings Citation Index, OAIster, Open-Grey, Canadian Electronic Library, and Canadian Research Index. Further, eligible studies will be searched on organizational websites such as International MARCE Society for Perinatal Mental Health, Pregnancy and Infant Loss Network (PAIL), Pregnancy After Loss Support (PALS), WHO Partnership for Maternal, Newborn and Child Health, Pregnancy Loss and Infant Death Alliance (PLIDA), International Stillbirth Alliance (ISA), and Canadian Association of Perinatal and Women’s Health Nurses (CAPWHN).

The search strategy will include literature that is not limited by language, publication year, publication status, or methodological quality. Articles retrieved through the search strategy that are not in English will be excluded during the study selection process. Qualitative studies will be excluded, as will other publication types such as books, book chapters, discussion papers, editorials, commentaries, letters, abstracts, posters, reviews, guidelines, and case studies.

From the included full-text articles, backward and forward citation searches will be conducted to create a final list of articles that meet the criteria.

### Data collection

#### Data management

EndNote X8 will be utilized to manage articles by removing duplicates, categorizing studies, and retrieving and storing full-text sources.

#### Study selection

The Microsoft Excel spreadsheet application will be used to facilitate organization of information throughout the study selection process. There will be three reviewers involved in the process, the author will be the primary reviewer (EMC), a fellow PhD candidate will be the secondary reviewer (KSB), and the supervisor of the first two reviewers will be the third reviewer (DEK). Initially, a training and calibration exercise to pilot the screening tool using the inclusion and exclusion criteria on approximately 30 titles and abstracts will be conducted with revisions made to the tool, as necessary. The titles and abstracts of the list of articles (level 1) will be screened independently by the primary and secondary reviewers with discrepancies resolved by the third reviewer. The articles selected based on their title and/or abstract will be retrieved in full text (level 2) and screened independently by the same two reviewers with discrepancies resolved by the third reviewer. The PRISMA flow diagram will be used to document the study selection process.

#### Data extraction

Data will be extracted from full-text articles using a data extraction template developed for the randomized controlled trial (RCT) study design and the non-randomized study design using Microsoft Excel. These templates will be customized to capture additional data specific to the intent of the review questions. A calibration exercise will be conducted to pilot the customized templates with five percent of included studies, and revisions will be made as necessary. The process of data extraction will begin with the primary reviewer (EMC) extracting and the secondary reviewer (KSB) verifying the accuracy of the data extracted. Discrepancies will be discussed, and if there is no consensus between the first two reviewers, consultation with the third reviewer (DEK) will provide resolution. To ensure comprehensive data is attained during data extraction, intervention protocols will be accessed. Table 1 outlines the data items that will be extracted from full-text articles.

**Table 1:** Data items to be extracted from included studies

Category	Data to be extracted
Study characteristics	First author, year, country, study objective, and study design
Recruitment	Recruitment strategy, sample size, group assignment: unit (individual, group, community), method (non/randomization), and bias minimization
Participant details	Eligibility criteria, demographics, mental illness history/diagnosis, perinatal loss (definition, type, time since loss, previous/repeated loss), pregnancy status, participation, and attrition
Measurement	Tool used, timing and frequency of assessments, method and setting of data collection, data collectors (who, training), confounders, and reliability/validity estimate for measurement tool
Intervention characteristics	Type, unit (individual, group), content of psychotherapeutic intervention (structure, objectives, goals), facilitator and credentials, delivery method (in-person, phone, online, distance), setting, timing of intervention initiation, number, frequency, length and duration of intervention, adherence (activities to enhance adherence, assessment of adherence or fidelity), materials (physical or information), tailoring, modifications (unplanned), and comparison group intervention
Outcomes	Duration and severity of depressive, anxiety, grief, and posttraumatic stress symptoms, changes in perception of support and care, coping, and adjustment

#### Assessment of methodological quality and risk of bias

Methodological quality and risk of bias will be assessed for each study individually by the primary (EMC) and secondary (KSB) reviewers with discrepancies resolved by the third reviewer (DEK). For the randomized studies included, the Cochrane Risk of Bias assessment tools for RCTs (RoB2) will be used to assess for bias created from the process of randomization, assignment and adherence to intended interventions, missing outcome data, outcome measurement, and selection of reported results [56]. The RoB2 ratings within each domain will be classified as low risk, high risk, and some concerns [56]. For the non-randomized studies included, the Risk of Bias in Non-Randomised Studies of Interventions (ROBINS-1) will be used to assess bias due to confounding, selection of participants, classification of interventions, deviations from intended interventions, missing data, measurement of outcomes, selection of the reported result, and overall bias [57]. The ROBINS-1 ratings within each domain will be classified as low risk, moderate risk, serious risk, critical risk, and no information [57]. Further, an assessment of intervention fidelity will be added to the RoB2 for RCT’s “other” category [56] and to the ROBINS-I’s “deviations from intended interventions” category [57].

### Data synthesis and analysis

Synthesis of the extracted data will be conducted in accordance with the York’s Centre for Reviews and Dissemination (CRD) guidelines [58]. A narrative synthesis will be used to aggregate studies by the validated measurement tool used and/or intervention type received to compare relationships within the data. The narrative synthesis process is intended to synthesize findings from the included studies, describe patterns within the studies, explore relationships within the results, examine factors impacting intervention effectiveness and effects, and assess robustness and generalizability [59].

#### Missing data

In the event there is missing data, an attempt to contact authors of the studies will be made. In addition, the attrition rates for each included study will be noted or calculated. If missing data is not obtained or if a study’s attrition rates are high, imputation of missing values will be performed. A sensitivity analysis will then be conducted by removing studies individually to determine the impact that each included study has on the overall intervention effect.

#### Assessment of heterogeneity

Since clinical and epidemiological heterogeneity is expected a priori, meta-analyses will be conducted using the random effects model where appropriate. The random effects model assumes the treatment effects follow a normal distribution, considering both within-study and between-study variation [60]. Forest plots will be used to visualize pooled estimates and the extent of heterogeneity among studies. We will quantify statistical heterogeneity by estimating the variance between studies using the *I*^2^ statistic. The *I*^2^ statistic is the proportion of variation in prevalence estimates that is due to genuine variation in prevalence rather than sampling (random) error [60]. The *I*^2^ statistic ranges between 0 and 100% (with values of 0–25% and 75–100% taken to indicate low and considerable heterogeneity, respectively) [61]. We will also report Tau2 and Cochran Q test with a *P* value of < 0.05 considered statistically significant (heterogeneity). Further, if a small number of studies limit the information available to adequately apply the random effects model, a fixed effect model or a Bayesian approach will be appropriate.

#### Assessment of meta-bias

If there are ten or more appropriate studies in the meta-analysis, meta-bias (reporting or publication) will be assessed by visualizing the funnel plot for each outcome which will be created from each study’s effect estimate and its study size [62]. Conducting Egger’s test of the intercept will quantify the funnel plot’s asymmetry [62].

#### Analysis of subgroups

With enough information from the included studies, a subgroup analysis will be conducted. The subgroup analysis will consider details related to intervention (type, content, facilitator, delivery method, setting, timing, frequency, length, duration), perinatal loss (type, previous losses), participant (present and past psychiatric condition, pregnancy status), and study design (RCT, quasi-experimental).

#### Confidence in cumulative evidence

The Grading of Recommendations Assessment, Development and Evaluation (GRADE) approach [63], as recommended in the Cochrane Handbook for Systematic Reviews of Interventions [60], will be used to assess the quality of the evidence for each outcome related to psychological distress, perception, coping, and adjustment. The intention in using GRADE is to increase confidence in the review’s cumulative findings which may be used to guide research in the future. The GRADE includes assessments on study design and quality, and consistency and directness and may be rated as high, moderate, low, and very low [63]. The factors that may downgrade the quality of the evidence include study quality limitations, study design, risk of bias, inconsistency, indirectness (not generalizable), imprecision (sparse data), and publication bias [63]. The factors assessed that may upgrade the quality of the evidence include large magnitude of effects, dose-response effect, and effect of all plausible factors [63, 64].

## Discussion

This protocol outlines the strategy that will be used to complete a systematic review and meta-analysis on the effectiveness of psychotherapeutic intervention on psychological distress, perception, coping, and adjustment in women who have experienced PL. The content and delivery method associated with effective psychotherapeutic interventions will be identified as well.

While there are a few individual studies that provide data related to the effectiveness of specific psychotherapeutic interventions on women affected by PL [37–39], there is no evidence in the literature of a comprehensive comparison of these intervention studies. The knowledge generated from this review will enhance existing evidence and may be used to develop new psychological intervention programs or to refine existing psychotherapy in effort to improve the quality of the services accessible to women after PL. This knowledge is especially important for women residing in medium- and low-resource settings, where access to treatment is likely to be significantly lower than in high-resource settings. With an improvement in relevant services, women will experience improved opportunity to recover after PL, reduced psychological distress, and enhanced resilience. To the best of the author’s knowledge, this will be the first systematic review that overtly and fully intends to generate evidence that can inform researchers and policy makers in expanding on related research and developing tailored interventions or programs that will improve outcomes for women affected by PL and their families.

## Supplementary information

**Additional file 1.** The Preferred Reporting Items for Systematic Reviews and Meta-Analysis Protocols (PRISMA-P) Checklist: Completed PRISMA-P checklist specific to this protocol.

**Additional file 2.** Search Strategy. Completed search strategy corresponding with this protocol.

## Data Availability

All results acquired within this review will be available by request through the corresponding author. Results may include database searches, search results, quality appraisal, and data extraction results from included studies.

## Abbreviations

CENTRAL: Cochrane Central Register of Controlled Trials
RCT: Randomized controlled trial
PROSPERO: International Prospective Register of Systematic Reviews
PL: Perinatal loss
PTS: Post-traumatic stress
CBT: Cognitive behavioral therapy
IPT: Interpersonal psychotherapy
PRISMA-P: Preferred Reporting Items for Systematic Review and Meta-Analysis Protocols
PRISMA: Preferred Reporting Items for Systematic Reviews and Meta-Analyses
PICOSS: Participants, interventions, comparators, outcomes, study designs, setting
PAIL: Pregnancy and Infant Loss Network
PALS: Pregnancy After Loss Support
WHO: World Health Organization
PLIDA: Pregnancy Loss and Infant Death Alliance
ISA: International Stillbirth Alliance
CAPWHN: Canadian Association of Perinatal and Women’s Health Nurses
RoB2: Cochrane Risk of Bias assessment for RCTs
ROBINS-1: Risk of Bias in Non-Randomized Studies of Interventions
CRD: York’s Centre for Reviews and Dissemination
GRADE: The Grading of Recommendations, Assessment, Development and Evaluation
