# Supplementary material for: Effectiveness of psychotherapeutic interventions on psychological distress in women who have experienced perinatal loss: a systematic review protocol
Source: Syst Rev. 2020 Jun 2;9:125. doi: 10.1186/s13643-020-01387-6 (PMC7268676; doi:10.1186/s13643-020-01387-6)
Supplement: Supplementary file 2 — Additional file 2. Search Strategy. Completed search strategy corresponding with this protocol. [file 13643_2020_1387_MOESM2_ESM.docx]

**FINAL SEARCH STRATEGIES**

**PERINATAL LOSS AND PSYCHOTHERAPY**

**JANUARY 29-30, 2019**

Database(s): **PsycINFO**1806 to January Week 3 2019 
Search Strategy:

| **#** | **Searches** | **Results** |
| --- | --- | --- |
| 1 | exp SPONTANEOUS ABORTION/ | 801 |
| 2 | (Infant* adj2 (loss* or death* or demise*)).mp. | 1485 |
| 3 | (Pregnancy adj2 loss*).mp. | 497 |
| 4 | miscarriage*.mp. | 1164 |
| 5 | (spontaneous abortion* or recurrent abortion*).mp. | 989 |
| 6 | (stillbirth* or still-birth* or stillborn* or still-born*).mp. | 875 |
| 7 | ((perinatal or prenatal or antenatal or peri-natal or pre-natal or ante-natal or postnatal or post-natal) adj2 (loss* or death* or demise*)).mp. | 571 |
| 8 | ((fetal or foetal or fetus* or foetus* or neonatal or neo-natal or newborn* or new-born*) adj2 (loss* or death* or demise*)).mp. | 807 |
| 9 | ((intrapartum or intra-partum or antepartum or ante-partum or intrauterine or intra-uterine or in-utero or inutero) adj2 (loss* or death* or demise*)).mp. | 79 |
| 10 | or/1-9 | 4688 |
| 11 | exp COUNSELING/ | 75116 |
| 12 | exp PSYCHOTHERAPY/ | 210035 |
| 13 | exp Cognitive Behavior Therapy/ | 18952 |
| 14 | exp Cognitive Therapy/ | 13138 |
| 15 | exp Behavior Therapy/ | 19256 |
| 16 | exp Interpersonal Psychotherapy/ | 1283 |
| 17 | exp "Acceptance and Commitment Therapy"/ | 1539 |
| 18 | exp Dialectical Behavior Therapy/ | 1108 |
| 19 | exp Couples Therapy/ | 4248 |
| 20 | exp Marriage Counseling/ | 5155 |
| 21 | exp Rational Emotive Behavior Therapy/ | 1765 |
| 22 | exp Guided Imagery/ | 718 |
| 23 | exp MINDFULNESS/ | 8447 |
| 24 | exp Family Therapy/ | 21406 |
| 25 | counsel*.mp. | 120226 |
| 26 | (psychotherap* or psycho-therap*).mp. | 178589 |
| 27 | (cognitive adj2 (therap* or psychotherap* or psycho-therap*)).mp. | 39896 |
| 28 | (acceptance adj1 commitment therap*).mp. | 311 |
| 29 | (acceptance-based adj1 therap*).mp. | 68 |
| 30 | (dialectical adj2 (therap* or psychotherap* or psycho-therap*)).mp. | 1976 |
| 31 | ((behavior* or behaviour* or interpersonal or inter-personal) adj1 (therap* or psychotherap* or psycho-therap*)).mp. | 48975 |
| 32 | (Functional Analytic adj (psychothera* or psycho-therap* or therap*)).mp. | 232 |
| 33 | Integrative Behavio* Couple* Therap*.mp. | 132 |
| 34 | ((grief or bereavement) adj2 (therap* or counsel*)).mp. | 1073 |
| 35 | (complicated grief adj (therap* or psycotherap* or psycho-therap*)).mp. | 7 |
| 36 | ((metacognitive or meta-cognitive) adj2 (therap* or counsel*)).mp. | 277 |
| 37 | (rational adj1 (emotive* or therap* or psychotherap* or psycho-therap*)).mp. | 2792 |
| 38 | guided imagery.mp. | 1503 |
| 39 | (mindfulness or mindfulness-based).mp. | 12046 |
| 40 | ((couple* or marital or family) adj2 (therap* or counsel*)).mp. | 35564 |
| 41 | (emotion focus* adj1 therap*).mp. | 747 |
| 42 | (IPT or CBT).mp. | 13529 |
| 43 | therap*.tw,id. | 393334 |
| 44 | or/11-43 | 605641 |
| 45 | 10 and 44 | 738 |

Database(s): **Ovid MEDLINE(R) and Epub Ahead of Print, In-Process & Other Non-Indexed Citations and Daily**1946 to January 28, 2019 
Search Strategy:

| **#** | **Searches** | **Results** |
| --- | --- | --- |
| 1 | *Abortion, Spontaneous/ or *Abortion, Habitual/ | 15121 |
| 2 | *Stillbirth/ | 2611 |
| 3 | *Fetal Death/ | 9909 |
| 4 | *Perinatal Death/ | 871 |
| 5 | (Infant* adj1 (loss* or death* or demise*)).tw,kf. | 9380 |
| 6 | (Pregnancy adj1 loss*).tw,kf. | 6336 |
| 7 | miscarriage*.tw,kf. | 12620 |
| 8 | (spontaneous abortion* or recurrent abortion*).tw,kf. | 10747 |
| 9 | (stillbirth* or still-birth* or stillborn* or still-born*).tw,kf. | 14666 |
| 10 | ((perinatal or prenatal or antenatal or peri-natal or pre-natal or ante-natal or postnatal or post-natal) adj1 (loss* or death* or demise*)).tw,kf. | 5499 |
| 11 | ((fetal or foetal or fetus* or foetus* or neonatal or neo-natal or newborn* or new-born*) adj1 (loss* or death* or demise*)).tw,kf. | 20965 |
| 12 | ((intrapartum or intra-partum or antepartum or ante-partum or intrauterine or intra-uterine or in-utero or inutero) adj1 (loss* or death* or demise*)).tw,kf. | 2854 |
| 13 | or/1-12 | 78833 |
| 14 | *Counseling/ | 15377 |
| 15 | *Psychotherapy/ | 35099 |
| 16 | behavior therapy/ or emotion-focused therapy/ or person-centered psychotherapy/ or psychotherapy, brief/ or psychotherapy, multiple/ or psychotherapy, psychodynamic/ or psychotherapy, rational-emotive/ | 31229 |
| 17 | cognitive behavioral therapy/ or "acceptance and commitment therapy"/ or mindfulness/ | 24900 |
| 18 | Dialectical Behavior Therapy/ | 7 |
| 19 | couples therapy/ or marital therapy/ | 2062 |
| 20 | Family Therapy/ | 8569 |
| 21 | counsel*.ti. | 19098 |
| 22 | (psychotherap* or psycho-therap*).tw,kf. | 44081 |
| 23 | (cognitive adj2 (therap* or psychotherap* or psycho-therap*)).tw,kf. | 19595 |
| 24 | (acceptance adj1 commitment therap*).tw,kf. | 30 |
| 25 | (acceptance-based adj1 therap*).tw,kf. | 21 |
| 26 | (dialectical adj2 (therap* or psychotherap* or psycho-therap*)).tw,kf. | 728 |
| 27 | ((behavior* or behaviour* or interpersonal or inter-personal) adj1 (therap* or psychotherap* or psycho-therap*)).tw,kf. | 22450 |
| 28 | (Functional Analytic adj (psychothera* or psycho-therap* or therap*)).tw,kf. | 40 |
| 29 | Integrative Behavio* Couple* Therap*.tw,kf. | 30 |
| 30 | ((grief or bereavement) adj2 (therap* or counsel*)).tw,kf. | 387 |
| 31 | (complicated grief adj (therap* or psycotherap* or psycho-therap*)).tw,kf. | 7 |
| 32 | ((metacognitive or meta-cognitive) adj2 (therap* or counsel*)).tw,kf. | 157 |
| 33 | (rational adj1 (emotive* or therap* or psychotherap* or psycho-therap*)).tw,kf. | 2632 |
| 34 | guided imagery.tw,kf. | 675 |
| 35 | (mindfulness or mindfulness-based).tw,kf. | 5921 |
| 36 | ((couple* or marital or family) adj2 (therap* or counsel*)).tw,kf. | 8191 |
| 37 | (emotion focus* adj1 therap*).tw,kf. | 113 |
| 38 | (IPT or CBT).tw,kf. | 11514 |
| 39 | or/14-38 | 160313 |
| 40 | 13 and 39 | 528 |

Database(s): **Embase**1974 to 2019 January 29 
Search Strategy:

| **#** | **Searches** | **Results** |
| --- | --- | --- |
| 1 | *spontaneous abortion/ | 9492 |
| 2 | *recurrent abortion/ | 3608 |
| 3 | *stillbirth/ or *perinatal death/ or *fetus death/ | 10121 |
| 4 | (Infant* adj1 (loss* or death* or demise*)).tw,kw. | 10944 |
| 5 | (Pregnancy adj1 loss*).tw,kw. | 10228 |
| 6 | miscarriage*.tw,kw. | 21751 |
| 7 | (spontaneous abortion* or recurrent abortion*).tw,kw. | 14584 |
| 8 | (stillbirth* or still-birth* or stillborn* or still-born*).tw,kw. | 19232 |
| 9 | ((perinatal or prenatal or antenatal or peri-natal or pre-natal or ante-natal or postnatal or post-natal) adj1 (loss* or death* or demise*)).tw,kw. | 7159 |
| 10 | ((fetal or foetal or fetus* or foetus* or neonatal or neo-natal or newborn* or new-born*) adj1 (loss* or death* or demise*)).tw,kw. | 26880 |
| 11 | ((intrapartum or intra-partum or antepartum or ante-partum or intrauterine or intra-uterine or in-utero or inutero) adj1 (loss* or death* or demise*)).tw,kw. | 3966 |
| 12 | or/1-11 | 97166 |
| 13 | *counseling/ | 15320 |
| 14 | *psychotherapy/ | 38112 |
| 15 | couple therapy/ or emotion-focused therapy/ or family therapy/ or guided imagery/ or marital therapy/ or mindfulness/ or rational emotive behavior therapy/ | 22063 |
| 16 | cognitive behavioral therapy/ | 7420 |
| 17 | *cognitive therapy/ or "acceptance and commitment therapy"/ or *behavior therapy/ | 28539 |
| 18 | counsel*.ti. | 23000 |
| 19 | (psychotherap* or psycho-therap*).tw,kw. | 57064 |
| 20 | (cognitive adj2 (therap* or psychotherap* or psycho-therap*)).tw,kw. | 29361 |
| 21 | (acceptance adj1 commitment therap*).tw,kw. | 44 |
| 22 | (acceptance-based adj1 therap*).tw,kw. | 32 |
| 23 | (dialectical adj2 (therap* or psychotherap* or psycho-therap*)).tw,kw. | 1018 |
| 24 | ((behavior* or behaviour* or interpersonal or inter-personal) adj1 (therap* or psychotherap* or psycho-therap*)).tw,kw. | 33218 |
| 25 | (Functional Analytic adj (psychothera* or psycho-therap* or therap*)).tw,kw. | 51 |
| 26 | Integrative Behavio* Couple* Therap*.tw,kw. | 30 |
| 27 | ((grief or bereavement) adj2 (therap* or counsel*)).tw,kw. | 516 |
| 28 | (complicated grief adj (therap* or psycotherap* or psycho-therap*)).tw,kw. | 10 |
| 29 | ((metacognitive or meta-cognitive) adj2 (therap* or counsel*)).tw,kw. | 213 |
| 30 | (rational adj1 (emotive* or therap* or psychotherap* or psycho-therap*)).tw,kw. | 3864 |
| 31 | guided imagery.tw,kw. | 971 |
| 32 | (mindfulness or mindfulness-based).tw,kw. | 7846 |
| 33 | ((couple* or marital or family) adj2 (therap* or counsel*)).tw,kw. | 12127 |
| 34 | (emotion focus* adj1 therap*).tw,kw. | 128 |
| 35 | (IPT or CBT).tw,kw. | 17054 |
| 36 | or/13-35 | 180702 |
| 37 | 12 and 36 | 744 |

Database(s): **EBM Reviews - Cochrane Central Register of Controlled Trials**December 2018 
Search Strategy:

| **#** | **Searches** | **Results** |
| --- | --- | --- |
| 1 | *Abortion, Spontaneous/ or *Abortion, Habitual/ | 17 |
| 2 | *Stillbirth/ | 0 |
| 3 | *Fetal Death/ | 0 |
| 4 | *Perinatal Death/ | 0 |
| 5 | (Infant* adj1 (loss* or death* or demise*)).tw,kf. | 250 |
| 6 | (Pregnancy adj1 loss*).tw,kf. | 457 |
| 7 | miscarriage*.tw,kf. | 1277 |
| 8 | (spontaneous abortion* or recurrent abortion*).tw,kf. | 447 |
| 9 | (stillbirth* or still-birth* or stillborn* or still-born*).tw,kf. | 521 |
| 10 | ((perinatal or prenatal or antenatal or peri-natal or pre-natal or ante-natal or postnatal or post-natal) adj1 (loss* or death* or demise*)).tw,kf. | 309 |
| 11 | ((fetal or foetal or fetus* or foetus* or neonatal or neo-natal or newborn* or new-born*) adj1 (loss* or death* or demise*)).tw,kf. | 971 |
| 12 | ((intrapartum or intra-partum or antepartum or ante-partum or intrauterine or intra-uterine or in-utero or inutero) adj1 (loss* or death* or demise*)).tw,kf. | 89 |
| 13 | or/1-12 | 3571 |
| 14 | *Counseling/ | 0 |
| 15 | *Psychotherapy/ | 2 |
| 16 | behavior therapy/ or emotion-focused therapy/ or person-centered psychotherapy/ or psychotherapy, brief/ or psychotherapy, multiple/ or psychotherapy, psychodynamic/ or psychotherapy, rational-emotive/ | 5073 |
| 17 | cognitive behavioral therapy/ or "acceptance and commitment therapy"/ or mindfulness/ | 646 |
| 18 | Dialectical Behavior Therapy/ | 0 |
| 19 | couples therapy/ or marital therapy/ | 214 |
| 20 | Family Therapy/ | 858 |
| 21 | counsel*.ti. | 3401 |
| 22 | (psychotherap* or psycho-therap*).tw,kf. | 5192 |
| 23 | (cognitive adj2 (therap* or psychotherap* or psycho-therap*)).tw,kf. | 9990 |
| 24 | (acceptance adj1 commitment therap*).tw,kf. | 449 |
| 25 | (acceptance-based adj1 therap*).tw,kf. | 11 |
| 26 | (dialectical adj2 (therap* or psychotherap* or psycho-therap*)).tw,kf. | 244 |
| 27 | ((behavior* or behaviour* or interpersonal or inter-personal) adj1 (therap* or psychotherap* or psycho-therap*)).tw,kf. | 9837 |
| 28 | (Functional Analytic adj (psychothera* or psycho-therap* or therap*)).tw,kf. | 5 |
| 29 | Integrative Behavio* Couple* Therap*.tw,kf. | 14 |
| 30 | ((grief or bereavement) adj2 (therap* or counsel*)).tw,kf. | 64 |
| 31 | (complicated grief adj (therap* or psycotherap* or psycho-therap*)).tw,kf. | 3 |
| 32 | ((metacognitive or meta-cognitive) adj2 (therap* or counsel*)).tw,kf. | 84 |
| 33 | (rational adj1 (emotive* or therap* or psychotherap* or psycho-therap*)).tw,kf. | 175 |
| 34 | guided imagery.tw,kf. | 376 |
| 35 | (mindfulness or mindfulness-based).tw,kf. | 2742 |
| 36 | ((couple* or marital or family) adj2 (therap* or counsel*)).tw,kf. | 1240 |
| 37 | (emotion focus* adj1 therap*).tw,kf. | 34 |
| 38 | (IPT or CBT).tw,kf. | 5921 |
| 39 | or/14-38 | 27976 |
| 40 | 13 and 39 | 57 |

SCOPUS Search January 29, 2019

( ( TITLE-ABS-KEY ( ( *psychotherap**  OR  *psycho-therap** ) ) )  OR  ( TITLE-ABS-KEY ( ( *cognitive*  W/2  ( *therap**  OR  *psychotherap**  OR  *psycho-therap** ) ) ) )  OR  ( TITLE-ABS-KEY ( ( *acceptance*  W/1  *commitment*  AND *therap** )  OR  ( *acceptance-based*  W/1  *therap** ) ) )  OR  ( TITLE-ABS-KEY ( ( *"complicated grief"*  OR  *"Functional Analytic"*  OR  *behavior**  OR  *behaviour**  OR  *interpersonal*  OR  *inter-personal*  OR  *dialectical* )  W/1  ( *therap**  OR  *psycotherap**  OR  *psycho-therap** ) ) )  OR  ( ( TITLE-ABS-KEY ( ( *rational*  W/1  ( *emotive**  OR  *therap**  OR  *psychotherap**  OR  *psycho-therap** ) ) )  OR  TITLE-ABS-KEY ( *"guided imagery"*  OR  *"Integrative Behavio* Couple* Therap*"* )  OR  TITLE-ABS-KEY ( ( *mindfulness*  OR  *mindfulness-based* ) )  OR  TITLE-ABS-KEY ( ( ( *couple**  OR  *marital*  OR  *family*  OR  *metacognitive*  OR  *meta-cognitive*  OR  *grief*  OR  *bereavement* )  W/2  ( *therap**  OR  *counsel** ) ) ) ) )  OR  ( TITLE-ABS-KEY ( ( *"emotion focus*"*  W/1  *therap** ) ) ) )  AND  ( ( ( TITLE-ABS-KEY ( *pregnancy*  W/2  *loss* )  OR  TITLE-ABS-KEY ( *miscarriage**  OR  *"spontaneous abortion*"*  OR  *"recurrent abortion"*  OR  *stillbirth**  OR  *still-birth**  OR  *stillborn**  OR  *still-born** )  OR  TITLE-ABS-KEY ( ( *infant**  W/2  ( *loss**  OR  *death**  OR  *demise** ) ) ) ) )  OR  ( ( TITLE-ABS-KEY ( ( ( *perinatal*  OR  *prenatal*  OR  *antenatal*  OR  *peri-natal*  OR  *pre-natal*  OR  *ante-natal*  OR  *postnatal*  OR  *post-natal* )  W/2  ( *loss**  OR  *death**  OR  *demise** ) ) )  OR  TITLE-ABS-KEY ( ( ( *fetal*  OR  *foetal*  OR  *fetus**  OR  *foetus**  OR  *neonatal*  OR  *neo-natal*  OR  *newborn**  OR  *new-born** )  W/2  ( *loss**  OR  *death**  OR  *demise** ) ) )  OR  TITLE-ABS-KEY ( ( ( *intrapartum*  OR  *intra-partum*  OR  *antepartum*  OR  *ante-partum*  OR  *intrauterine*  OR  *intra-uterine*  OR  *in-utero*  OR  *inutero* )  W/2  ( *loss**  OR  *death**  OR  *demise** ) ) ) ) ) )
